# Dietary Polyphenols Support *Akkermansia muciniphila* Growth via Mediation of the Gastrointestinal Redox Environment

**DOI:** 10.3390/antiox13030304

**Published:** 2024-02-29

**Authors:** Charlene B. Van Buiten, Valerie A. Seitz, Jessica L. Metcalf, Ilya Raskin

**Affiliations:** 1 Department of Food Science and Human Nutrition, College of Health and Human Sciences, Colorado State University, Fort Collins, CO 80525, USA; 2 Department of Animal Sciences, College of Agricultural Sciences, Colorado State University, Fort Collins, CO 80525, USA; valerie.lindstrom@colostate.edu (V.A.S.); jessica.metcalf@colostate.edu (J.L.M.); 3 Department of Plant Biology, School of Environmental and Biological Sciences, Rutgers University, New Brunswick, NJ 08901, USA; raskin@rutgers.edu

**Keywords:** polyphenol, reactive oxygen species, gut microbiome, metabolic syndrome, vitamin, antioxidant, *Akkermansia*, 16S rRNA amplicon

## Abstract

Obesity and metabolic dysfunction have been shown to be associated with overproduction of reactive oxygen species (ROS) in the gastrointestinal (GI) tract, which contributes to dysbiosis or imbalances in the gut microbiota. Recently, the reversal of dysbiosis has been observed as a result of dietary supplementation with antioxidative compounds including polyphenols. Likewise, dietary polyphenols have been associated with scavenging of GI ROS, leading to the hypothesis that radical scavenging in the GI tract is a potential mechanism for the reversal of dysbiosis. The objective of this study was to investigate the relationship between GI ROS, dietary antioxidants and beneficial gut bacterium *Akkermansia muciniphila*. The results of this study demonstrated *A. muciniphila* to be a discriminant microorganism between lean (*n* = 7) and obese (*n* = 7) mice. The relative abundance of *A. muciniphila* was also found to have a significant negative correlation with extracellular ROS in the GI tract as measured using fluorescent probe hydroindocyanine green. The ability of the dietary antioxidants ascorbic acid, β-carotene and grape polyphenols to scavenge GI ROS was evaluated in tandem with their ability to support *A. muciniphila* bloom in lean mice (*n* = 20). While the relationship between GI ROS and relative abundance of *A. muciniphila* was conserved in lean mice, only grape polyphenols stimulated the bloom of *A. muciniphila*. Analysis of fecal antioxidant capacity and differences in the bioavailability of the antioxidants of interest suggested that the poor bioavailability of grape polyphenols contributes to their superior radical scavenging activity and support of *A. muciniphila* in comparison to the other compounds tested. These findings demonstrate the utility of the GI redox environment as a modifiable therapeutic target in the treatment of chronic inflammatory diseases like metabolic syndrome.

## 1. Introduction

Metabolic syndrome (MetS) and its clinical markers (hyperglycemia, dyslipidemia, hypertension and obesity) affect 1 in 3 American adults and have continued to rise in prevalence over the last several decades [1]. MetS is associated with increased risk for developing chronic diseases including type 2 diabetes and cardiovascular disease [2]. Typically, MetS is managed through pharmaceutical intervention or lifestyle changes including dietary intervention. Though these interventions are typically aimed towards reducing energy intake [3], risk of MetS is inversely associated with fruit and/or vegetable intake [4]. This is thought to be due in part to the concurrent intake of fruit- and/or vegetable-associated compounds with antioxidant capacity including vitamins and phytochemicals [5].

Dietary intervention with antioxidants has been shown to improve metabolic health by modifying the gut microbiome. The gut microbiome comprises a dynamic ecology of microorganisms which are able to participate in biological crosstalk between hosts, diet and inflammation across a variety of chronic conditions (e.g., MetS, inflammatory bowel disease) [6,7]. In instances of malnutrition, including obesity-associated over-nutrition, the gut microbiome has been shown to become dysregulated, leading to alterations in metabolic homeostasis and the stimulation of inflammatory responses [7]. These findings have motivated further investigation of dietary interventions which may improve systemic health outcomes by modifying the gut microbiome by supporting the growth of organisms associated with reduced inflammation of the gastrointestinal (GI) tract.

One such intervention is the oral intake of polyphenols from grapes and cranberries, which have been shown to stimulate beneficial gut anaerobe *Akkermansia muciniphila* [8,9,10]. *A. muciniphila* is known to be depleted in individuals with metabolic disorders including, but not limited to, obesity, type 2 diabetes and cardiovascular disease. Furthermore, *A. muciniphila* has been shown to regulate gut inflammation, glucose metabolism, and gut barrier function, all of which are compromised in the aforementioned disorders [11]. Presently, the precise mechanism by which dietary antioxidants influence metabolic outcomes and modify the gut microbiome, including the stimulation of *A. muciniphila* bloom, remains unclear. Better understanding of the link between dietary antioxidant intake and *A. muciniphila* may provide improved clinical insight towards targeted modification of the gut microbiome in the reversal of metabolic disorders.

One possible mechanism by which dietary antioxidants mediate MetS and the gut microbiome is through the modification of gut oxygen levels and scavenging of reactive oxygen species (ROS), which are overproduced by metabolically compromised individuals concomitantly with chronic, low-grade inflammation of the gastrointestinal tract [12]. Broadly, the term “ROS” comprises a group of chemically reactive molecules containing oxygen including superoxide anions, hydrogen peroxides, hydroxyl radicals, singlet oxygen, peroxyl radicals, and nitric oxide [13]. Overproduction of ROS leads to oxidative stress when ROS production outpaces endogenous antioxidant systems [14], and increased levels of oxygen in the GI tract has been associated with a reduction in the abundance of oxygen-intolerant microorganisms [15]. Notably, *A. muciniphila* is an oxygen-intolerant microorganism, suggesting a possible mechanistic link between the tendency for *A. muciniphila* to be underrepresented in the gut microbiome of individuals with conditions associated with oxidative stress.

Recently, a non-invasive method was developed for measuring ROS in the lumen of the GI tract of mice in situ [16]. In this method, mice are gavaged with hydroindocyanine green (hICG), a membrane-impermeable fluorescent dye which specifically reacts with superoxide (O_2_^•−^) and hydroxyl radicals (HO^•^) [17]. Upon exposure to extracellular O_2_^•-^/HO^•^ in the GI lumen, hICG becomes oxidized and generates fluorescent oxidation products which can be measured using near-infrared fluorescent (NIRF) imaging. Notably, this method is sensitive enough to detect nanomolar concentrations of O_2_^•−^/HO^•^, and the NIRF signal generated increases in intensity as a function of O_2_^•−^/HO^•^ concentration [17].

Using this method, we have shown that elemental iron, a potent oxygen scavenger, modifies the GI microenvironment upon administration to lean and obese mice. Scavenging O_2_^•−^/HO^•^ from the GI lumen, as measured by a decrease in NIRF signaling, led to the restructuring of the gut microbiome and improvement of metabolic health [18]. Luminal O_2_^•−^/HO^•^ scavenging has also been observed in common dietary antioxidants, including polyphenols [16]. This has led to the hypothesis that increases in *A. muciniphila* and improvements to metabolic health upon treatment with dietary polyphenols may be due to the mediation of luminal ROS. However, direct correlations between these characteristics have not been demonstrated in the literature, and it is unknown whether *A. muciniphila* bloom is unique to dietary polyphenols or occurs as a result of modification to the GI redox environment regardless of the dietary antioxidant administered.

This study aimed to determine the relationship between luminal O_2_^•−^/HO^•^, henceforth referred to as GI ROS, and markers of metabolic health including body weight, oral glucose tolerance and the gut microbiome in both lean and obese mice. Specifically, we sought to identify microorganisms that were both discriminatory between lean and obese mice and sensitive to the GI redox environment, as measured using NIRF signaling. We next investigated the plasticity of these markers as a function of diet by comparing the temporal effects of three dietary antioxidants (ascorbic acid, β-carotene, grape polyphenol extract) on GI ROS, fecal antioxidant activity and relative abundance of *A. muciniphila* in lean mice. The panel of dietary antioxidants chosen featured differing solubility and bioavailability, allowing for insight towards how these characteristics may contribute to efficacy in the modulation of the gut microbiome and gut redox status (Table 1). Our findings further define the relationship between gut oxygen status and metabolic status, and highlight luminal radical scavenging as a potential mechanism of action for dietary antioxidants to influence metabolic health.

## 2. Materials and Methods

### 2.1. Chemicals and Reagents

Indocyanine green was purchased from Sigma Aldrich (St. Louis, MO, USA). Conversion to hydroindocyanine green (hICG) was completed according to previously described methods using ethanol and sodium borohydride (Sigma Aldrich, St. Louis, MO, USA) [16,17,18,30]. L-ascorbic acid, β-carotene, 2,2′-azino-bis(3-ethylbensothiazolin-6-sulfonic acid) (ABTS), 6-hydroxy-2,5,7,8-tetramethylchroman-2-carboxylic acid (Trolox), and procyanidin B_2_ were purchased from Sigma Aldrich (St. Louis, MO, USA). Grape polyphenol extract (GPE) was produced according to previously described methods [16]. The total procyanidin content of GPE was determined to be 82.21 ± 3.98 mg procyanidin B_2_ equivalents per 100 mg based on the DMAC assay [31].

### 2.2. Identification of Relationships between Luminal ROS, Metabolic Health and the Gut Microbiome

#### 2.2.1. Animals and Protocols

Specific taxa that both differentiate lean/low-fat diet-fed and obese/high-fat diet-fed mice and correlate with markers of metabolic health including body weight, oral glucose tolerance and GI ROS were identified based on a secondary analysis of previously published data [18]. In brief, male C57Bl/6J mice (*n* = 14) were purchased from Jackson Labs (Bar Harbor, ME, USA) and had ad libitum access to either a low-fat diet (*n* = 7; 10% kcal from fat, D12450J; Research Diets, Inc., New Brunswick, NJ, USA) or a high-fat diet (*n* = 7; 60% kcal from fat; D12492; Research Diets, Inc., New Brunswick, NJ, USA), prior to being assigned to a treatment group which dictated when the evaluation of metabolic status would take place. All mice were housed at a constant temperature on a 12 h light/dark cycle with ad libitum access to water and their assigned diet. The mice included in this study represent the unique subset of mice that underwent NIRF imaging, oral glucose tolerance (OGT) testing and fecal collection prior to beginning the daily administration of treatments in the previous study [18]. Thus, the mice included in this study represent only lean and obese mice with no additional treatment. Though mice were housed in groups of 3, each mouse in this study represents a unique cage. For ease of comparison between data sets, the methods described below for NIRF imaging and OGT testing are identical to those used in the previously published study.

#### 2.2.2. Analysis of Microbial DNA

The comparison of the gut microbiota of lean vs. obese mice in relation to near infrared fluorescence signaling was conducted using previously published data wherein the relationship between specific microbial taxa and GI ROS was not defined [18]. Raw sequence reads were accessed from the publicly available data set (NCBI BioProject ID PRJNA655513). Reads were demultiplexed using QIIME2 (2023.5) [32]. Using the DADA2 denoise-single command (--p-trim-left 19, --ptrunc-len 215) [33], demultiplexed reads were denoised to produce an amplicon sequence variant (ASV) table and filtered. A total of 14 fastq files were reanalyzed, resulting in a total 439,642 reads with an average of more than 26,000 reads per sample (Supplementary File S1). After denoising, 553 ASVs were detected. Feature classification was completed by comparing the ASV table against the Silva v138.1 database [34] using the q2-feature-classifier plugin [35] with the classify-sklearn method [36].

### 2.3. Influence of Dietary Supplementation of GI ROS and A. muciniphila Bloom

#### 2.3.1. Animals and Protocols

In total, 20 male C57Bl/6J mice were purchased from Jackson Labs (Bar Harbor, ME, USA) at 5 weeks of age. All mice were housed at a constant temperature on a 12 h light/dark cycle with ad libitum access to water and a low-fat diet (10% kcal from fat; D12450J; Research Diets, Inc., New Brunswick, NJ, USA). Mice were acclimated to single housing for one week prior to treatment administration. All animal procedures were undertaken with the approval of Rutgers University’s Institutional Animal Care and Use Committee (Protocol: PROTO999900260).

After housing acclimation, all mice were randomly assigned to a treatment group: control, ascorbic acid, β-carotene or grape pomace extract. Mice were treated with their assigned dietary antioxidant or vehicle control (water) via oral gavage (360 mg/kg body weight) daily for 14 days before imaging. Fecal samples were collected from each mouse on days 0, 7 and 14 for measurement of total bacterial load and relative abundance of *A. muciniphila* using qPCR. Antioxidant activity was measured for fecal samples collected on days 0 and 14 using the ABTS assay [37].

#### 2.3.2. Evaluation of Metabolic Status

The body weight of each mouse was recorded in grams (g). OGT was determined using the Clarity BG1000 Blood Glucose Monitoring System (Clarity Diagnostics, Boca Raton, FL, USA). Animals were fasted for 6 h. Fasting blood glucose was measured, and then all mice were gavaged with 2 g glucose per kg body weight. OGT was calculated as the area under the curve for blood glucose testing 0.5, 1, 2 and 3 h after glucose administration.

#### 2.3.3. Near-Infrared Fluorescence Imaging of GI ROS

Imaging of GI ROS in situ was performed as previously described [18,38]. In brief, abdominal hair was removed from mice 24 h before imaging by shaving and through application of a commercial depilatory (Church & Dwight, Ewing Township, NJ, USA). Animals were gavaged with hICG (2 g/kg body weight) one hour before being anesthetized with 3% isoflurane for imaging. Mice were placed ventrally on the imaging stage of an In Vivo MS FX PRO imaging system (Bruker, Ettlingen, Germany) for the acquisition of brightfield and infrared images (Ex. 760 nm, Em. 830 nm) of the abdominal area. The fluorescent intensity was quantified using Bruker Molecular Imaging Software. For each image, the background intensity was set to zero, and the fluorescent range was normalized to the range of 9.0 × 10^4^ to 8.0 × 10^6^ photons/s/mm^2^. Identical elliptical regions of interest were drawn onto each fluorescent image (117.43 × 159 pixels; interior area = 14,627) in order to express fluorescence intensity for each image in photons/s/mm^2^. After imaging, mice were sacrificed via CO_2_ asphyxiation. The fat pads, liver and cecum from each mouse were collected and weighed.

#### 2.3.4. Fecal Antioxidant Activity

The antioxidant activity of fecal samples collected from mice on day 0 and day 14 of the study was measured using the ABTS assay. In brief, frozen fecal samples were mixed with 50% ethanol at a rate of 100 mg feces/1 mL, and then they were homogenized with a Geno/Grinder (Model 2010, Metuchen, NJ, USA) at 1500 rpm for 8 min. The samples were then centrifuged at 13,000 rcf for 40 min. The supernatant was reserved for analysis using the ABTS assay according to the previously established methods [37]; the absorbance was measured at 734 nm on a BioTek Synergy HT Multi-Detection Plate Reader (BioTek, Winooski, VT, USA) and antioxidant activity was expressed in terms of Trolox equivalents based on a standard curve of 0–600 μg/mL in 95% ethanol.

#### 2.3.5. Extraction and Quantification of Microbial DNA

To understand the influence of dietary antioxidants on *A. muciniphila* bloom, bacterial DNA was extracted from frozen fecal samples using the PowerSoil bacterial DNA extraction kit (Qiagen, Hilden, Germany) and cleaned using Nucleospin gDNA cleanup columns (Machery-Nagel, Düren, Germany). The DNA concentration and purity were calculated using a Nanodrop spectrophotometer (Nanodrop Technologies, Wilmington, DE, USA). The samples were diluted to 2.5 ng/μL for the quantitation of *A. muciniphila* (AM1: 5′CAGCACGTGAAGGTGGGGAC, AM2: 5′CCTTGCGGTTGGCTTCAGAT) relative to the total bacterial load using qPCR (U341F: 5′CCTACGGGRSGCAGCAG, U515R: 5′TACCGCGGCKGCTGRCAC) [39]. All qPCR reactions were performed using Power SYBR Green PCR Master Mix and the QuantStudio 3 Real-Time PCR System (Applied Biosystems, Waltham, MA, USA). The relative abundance of *A. muciniphila* was calculated as the percentage of the total bacteria load.

### 2.4. Statistical Analysis

The alpha diversity of microbial communities was calculated using the diversity function from the vegan package [40] in R (v4.0.2) [41]. The beta diversity was estimated using the weighted unifrac distance matrix; principal coordinates analysis was run in QIIME2 [32]. The compositional differences across treatments were quantified using mrpp with an ANOVA model in R. The differences between communities across treatments were quantified using PERMANOVA. The LEfSe analysis was performed at the α value of 0.05 for the Kruskal–Wallis test, and the threshold of 2 on the logarithmic LDA score for discriminative features (Supplementary File S1) [42]. The phenotypic data were analyzed in Prism 10; the specific statistical tests are noted in figure captions. Results were considered significant at *p* ≤ 0.05. 

## 3. Results

### 3.1. Intraluminal Redox Status Strongly Correlates with Metabolic Status

**Figure 1:**
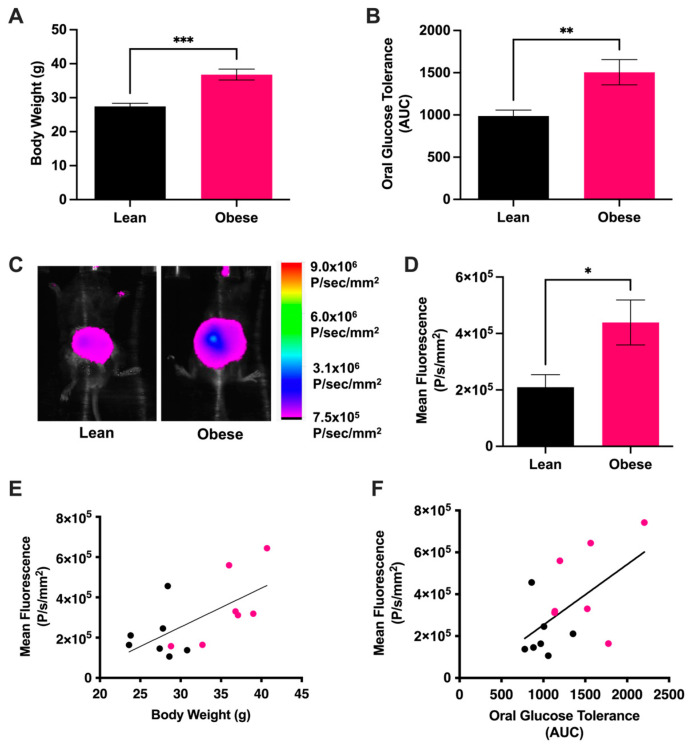
ROS-associated NIRF signaling strongly correlates with metabolic status as determined with oral glucose tolerance. (**A**) Mice fed a high-fat diet demonstrated greater body weight than mice fed a low-fat diet, allowing mice to be differentiated based on the lean vs. obese phenotype. Data were analyzed using an unpaired *t*-test and are reported as mean ± SD; *n* = 7 mice per group, *** *p* ≤ 0.001. (**B**) Oral glucose tolerance, calculated as area under the curve (AUC) over the course of 3 h. Data were analyzed using an unpaired *t*-test and are reported as mean ± SD; *n* = 7 mice per group, ** *p* ≤ 0.01. (**C**) Representative overlay NIRF and corresponding brightfield images of lean and obese mice. The NIRF intensity scale, shown at right, was normalized using Bruker Molecular Imaging Software. (**D**) ROS-associated NIRF in lean and obese mice. Data were analyzed using an unpaired *t*-test and are reported as mean ± SD; *n* = 7 mice per group, * *p* ≤ 0.05. (**E**) Pearson correlation of NIRF signaling with body weight. Black points denote lean mice while pink points denote obese mice; *n* = 14 mice, *r* = 0.6405, *p* = 0.00136. (**F**) Pearson correlation of NIRF signaling with oral glucose tolerance. Black points denote lean mice while pink points denote obese mice; *n* = 14 mice, *r* = 0.5712, *p* = 0.0329.

Obese mice fed a high-fat diet were shown to have significantly greater body weight than lean mice fed a low-fat diet (Figure 1A). The metabolic status of the mice was assessed by measuring oral glucose tolerance (OGT); as expected, the obese mice demonstrated higher blood glucose levels (Figure 1B). Stationary NIRF imaging of lean and obese mice demonstrated that the levels of extracellular O_2_^•−^/HO^•^ in the GI lumen, henceforth referred to as GI ROS, was over two-fold greater in obese mice than in lean mice (Figure 1C,D). GI ROS was significantly correlated with body weight (Figure 1E) and OGT (Figure 1F), suggesting that mediation of GI ROS may be a strategy for improving metabolic health.

### 3.2. Mouse Phenotype Influences Microbial Community Structure

**Figure 2:**
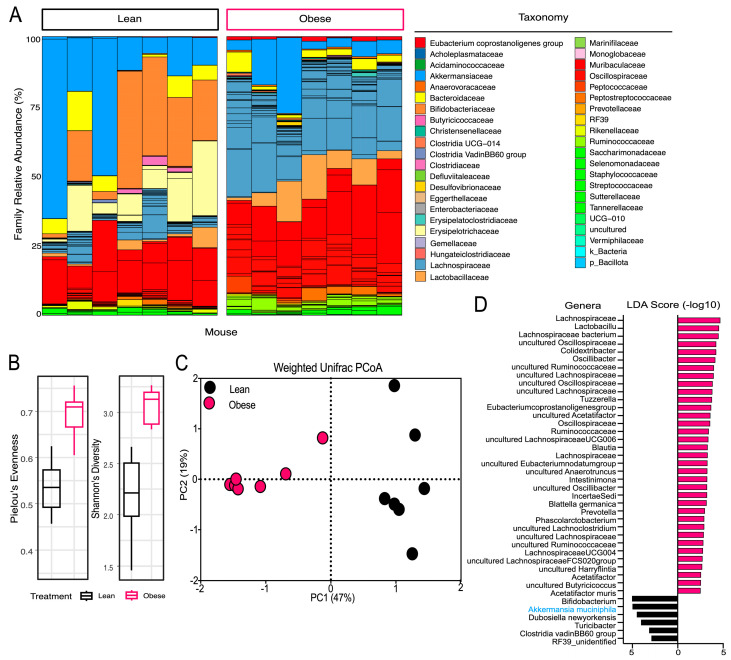
16S rRNA gene diversity metrics and membership differ significantly based on mouse phenotype. (**A**) Taxa bar plots highlight taxonomic differences between genotypes, summed at the family level, according to relative abundance. Obese (*n* = 7) and lean (*n* = 7) mice showed different microbial community profiles at the family level. (**B**) Boxplots show two alpha diversity metrics: Pielou’s evenness (left) and Shannon’s diversity index (right) for each phenotype. Box center displays the median, the end of the box shows the upper and lower quartiles, and whiskers show the minimum and maximum values. Color corresponds to genotype. (**C**) Principal coordinate analysis of the weighted unifrac distance metric shows differences in microbial community structure between phenotypes. Principal coordinate (PC) 1 accounts for 47% of variation observed, while PC2 accounts for 19% of variation. (**D**) Linear discriminant effect size (LEfSe) analysis found that 41 ASVs were discriminant in either phenotype (*p* < 0.05). Taxonomy is listed by genus, unless a higher taxonomy level was assigned. Size of bar represents linear discriminant analysis (LDA) score and direction, and color indicates which phenotype the ASV was discriminant in.

16S rRNA gene amplicons were used to profile the fecal microbial diversity and membership across lean and obese C57Bl/6J mice, resulting in 553 amplicon sequencing variants (ASVs) representing eight phyla and 43 families (Figure 2A). Diversity analyses revealed significantly greater alpha diversity (Shannon’s) and evenness (Pielou’s) in the microbiota of the obese mice compared to the lean mice (Figure 2B). A significant difference in the composition of the fecal microbiomes based on the phenotype was also observed (Figure 2C). To more specifically resolve which organisms were responsible for differentiating phenotypes, linear discriminant effect size (LEfSe) analysis was performed [42]. In total, LefSe identified 41 discriminant taxa. A total of 35 discriminant genera were identified in the obese phenotype, while only 6 genera were identified as discriminant for the lean phenotype (Figure 2D). The six lean–discriminant genera included members of *Bacillota* (e.g., *Turicibacter*, *Dubosiella newyorkensis*, *Clostridia vadinBB60* group and an unidentified *Bacilli*, RF39_unidentified), *Actinobacteriota* (e.g., *Bifidobacterium*) and *Verrucomicrobiota* (e.g., *A. muciniphila*) phyla.

### 3.3. Level of GI ROS, OGT Is Inversely Correlated with Relative Abundance of the Beneficial Anaerobe Akkermansia muciniphila

**Figure 3:**
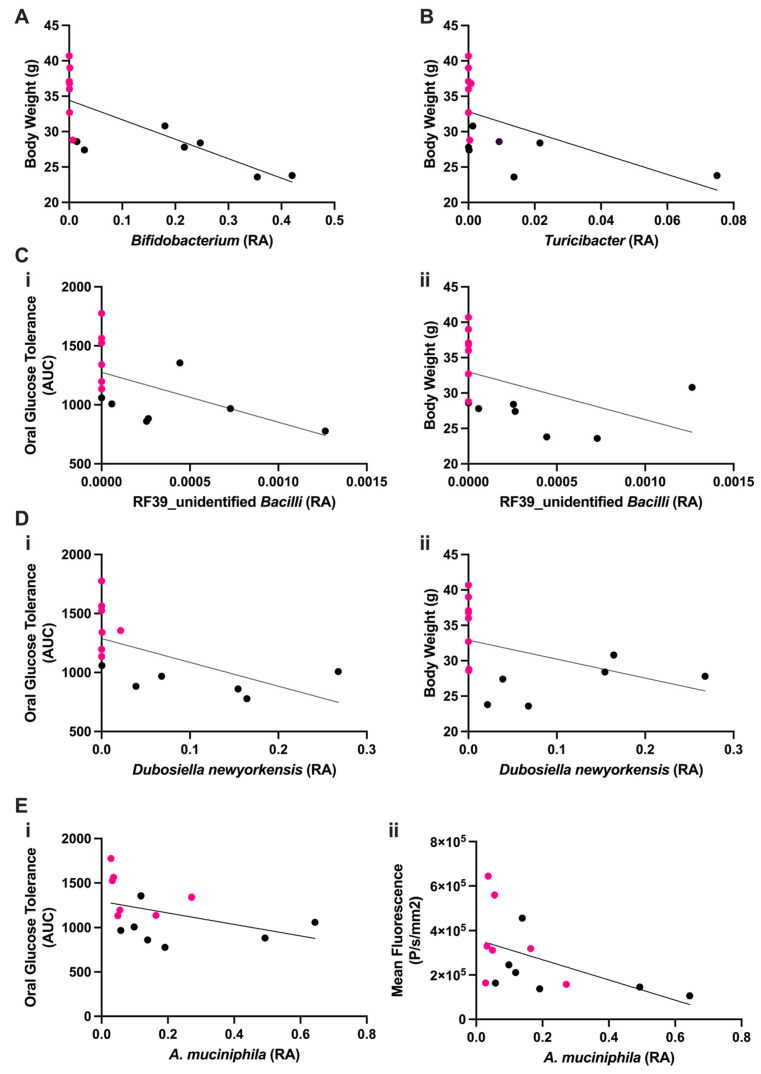
Discriminant taxa are negatively associated with body weight and oral glucose tolerance and ROS-associated NIRF. (**A**) *Bifidobacterium* is negatively associated with body weight; *r* = −0.8857, *p* < 0.0001. (**B**) *Turibacter* is negatively associated with body weight; *r* = −0.6565, *p* = 0.0133. (**C**) An unidentified Bacilli was found negatively correlated with (i) oral glucose tolerance (*r* = −0.662, *p* = 0.0122) and (ii) body weight (*r* = −0.7326, *p* = 0.0042). (**D**) *Dubosiella newyorkensis* is negatively correlated with (i) oral glucose tolerance (*r* = −0.7549, *p* = 0.0029) and (ii) body weight (*r* = −0.7732, *p* = 0.002). (**E**) *A. muciniphila* is negatively correlated with (i) oral glucose tolerance (*r* = −0.6088, *p* = 0.0236) and (ii) ROS-associated NIRF (*r* = −0.6308, *p* = 0.0181). All relationships were tested using Spearman’s correlation with *n* = 14 mice. Black points denote lean mice, while pink points denote obese mice; RA = relative abundance.

The relationships between the six lean-associated discriminatory taxa identified in the mouse fecal microbiomes and metabolic status was determined by identifying correlations between the relative abundance of each fecal bacteria ASV from each mouse to the corresponding phenotype measurements including body weight, OGT and GI ROS. Of the three phenotypic markers, negative correlations were observed between *Turicibacter*, *Bifidobacterium* and body weight (*p* < 0.01) (Figure 3A,B). *D. newyorkensis* and an unidentified *Bacilli* (RF_39 unidentified) were negatively correlated with body weight and OGT (*p* < 0.05) (Figure 3C,D). Interestingly, *A. muciniphila* was negatively associated with OGT (*p* < 0.05) and was the only microorganism demonstrating a significant negative correlation with GI ROS (*p* < 0.05) (Figure 3E).

### 3.4. Physical and Chemical Properties of Antioxidants Influence Intraluminal ROS and Akkermansia muciniphila Bloom

**Figure 4:**
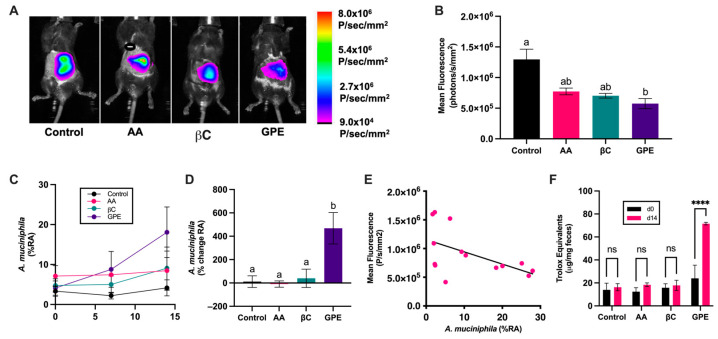
Dietary antioxidants differentially affect GI ROS and *A. muciniphila* growth. (**A**) ROS-associated NIRF signaling of lean mice after 14 days of treatment with dietary antioxidants demonstrates that GPE significantly reduces GI ROS. The NIRF intensity scale, shown at right, was normalized using Bruker Molecular Imaging Software. Data were analyzed using one-way ANOVA with Tukey’s multiple comparisons test and are reported as mean ± SD; *n* = 5 mice per group; Control vs. AA, *p* = 0.0582; Control vs. βC, *p* = 0.0584; Control vs. GPE, *p* = 0.0117. Values not sharing common letters are significantly different from one another (*p* ≤ 0.05). (**B**) Representative overlay NIRF and corresponding brightfield images of lean mice treated with dietary antioxidants for 14 days. (**C**) Change in relative abundance of *A. muciniphila* over the course of 14 days of treatment with dietary antioxidants. No significant differences in raw values were observed across time or between treatments. Data were analyzed using two-way ANOVA with Tukey’s multiple comparisons test and are reported as mean ± SD. Values not sharing common letters are significantly different from one another (*p* ≤ 0.05). (**D**) Relative changes in *A. muciniphila* relative abundance demonstrates that GPE stimulates bloom in comparison to the control, while the other dietary antioxidants do not. Data were analyzed using one-way ANOVA with Tukey’s multiple comparisons test and are reported as mean ± SD; *n* = 5 mice per group. Values not sharing common letters are significantly different from one another (*p* ≤ 0.05). (**E**) NIRF signaling is inversely correlated with *A. muciniphila* relative abundance after 14 days of treatment with dietary antioxidants; Spearman *r* = −0.6264, *p* = 0.0191. (**F**) Antioxidant activity (Trolox equivalents) in fecal samples before (d0) and after (d14) daily exposure to dietary antioxidants. Data were analyzed using two-way ANOVA with Tukey’s multiple comparisons test and are reported as mean ± SD; *n* = 5 mice per group, **** *p* ≤ 0.0001, ns = not significant. AA = ascorbic acid; βC = β-carotene; GPE = grape polyphenol extract.

To determine whether *A. muciniphila* bloom is based solely on reducing GI ROS or instead is unique to treatment with grape polyphenols, 20 lean male C57Bl/6J mice were treated with ascorbic acid, β-carotene, grape polyphenol extract (GPE) or water (vehicle control) for 14 days. GI ROS and OGT were measured 24 h after the final treatment, and fecal samples were collected to measure the relative abundance of *A. muciniphila*. Daily treatment with dietary antioxidants did not affect phenotypic characteristics including body weight, or weight of the liver, fat pads or cecum (Figure S1). Similarly, OGT was not affected, as the mice began the study metabolically healthy (Figure S2). Interestingly, each antioxidant treatment reduced ROS-associated NIRF in comparison to the control, but only treatment with GPE resulted in significantly lower levels of GI ROS (*p* = 0.0073); Figure 4A,B).

While the relative abundance of *A. muciniphila* demonstrated a trend towards increasing in both the β-carotene and GPE-treated mice, the variation between individual mice was such that these increases were not significant (Figure 4C). We considered *A. muciniphila* relative abundance from the perspective of increases relative to baseline abundance (Figure 4D), as it has been shown that grape proanthocyanidin-induced *A. muciniphila* bloom is dependent on baseline abundance [43]. Compared to the baseline, GPE induced an average of a 468.11% increase in relative abundance, which was significantly greater than the changes observed in the control group. Across all mice in the study, the relative abundance of *A. muciniphila* was inversely associated with ROS-associated NIRF (Figure 4E).

Fecal samples collected from mice on days 0 and 14 were analyzed for antioxidant activity using the ABTS assay (Figure 4F). Fecal antioxidant activity was not changed in the control, ascorbic acid or β-carotene groups, but a significant increase in antioxidant activity was observed as a result of GPE treatment.

## 4. Discussion

Previous studies have shown that dietary antioxidants can reduce extracellular ROS (O_2_^•−^/HO^•^) in the GI tract [16] and that these modifications to the GI redox environment may be linked directly to improvements in metabolic status and modification of the gut microbiome [18,44]. ROS scavenging has been hypothesized as a mechanism by which dietary supplementation with proanthocyanidins supports the growth of beneficial gut bacterium *A. muciniphila*, as *A. muciniphila* is an anaerobe. Indeed, modification of the redox environment has been shown to induce changes in gene expression of some bacterial species as well as their host [45,46], and scavenging intestinal ROS supports the growth of microorganisms without ROS defense systems [18]. In the present study, we tested the hypothesis that a relationship exists between the GI redox environment and *A. muciniphila*, and that scavenging ROS from the GI lumen would concomitantly support the growth of *A. muciniphila*.

To test this hypothesis, we first compared lean and obese mice on the basis of phenotypic markers including body weight, oral glucose tolerance and intestinal ROS levels, and the gut microbiota. Comparison of lean and obese mice demonstrated significant correlations between ROS-associated fluorescence and phenotypic markers (Figure 1), which further validates the use of this in situ method for evaluating metabolic health. Though this method has been used to compare lean and obese mice in other studies [16,18], the relationship between NIRF signaling and phenotypic markers for MetS had not previously been characterized.

An analysis of the gut microbiota revealed significant differences between the microbial communities of each group. Notably, the obese mice demonstrated greater alpha diversity and evenness than the lean mice. While greater diversity is typically associated with lean individuals, matched diets such as those used in this study do not allow the differences in fiber that are normally observed in the diets of lean versus obese individuals and contribute to increased diversity in the gut microbiome of lean individuals due to prebiotic effects [47]. With respect to specific microbial communities, total of 41 discriminant taxa were identified, including 35 obese–discriminant tax and 6 lean–discriminant taxa (Figure 2). Interestingly, one taxa of the *Prevotella* genus was discriminant, while the remaining 34 out of 35 obese–discriminant taxa were from the *Bacillota* phylum and primarily from the *Lachnospiraceae*, *Ruminococcaceae*, *Oscillospiraceae* families. This finding is consistent with the literature that obesity is generally associated with discordant shifts towards a *Bacillota*-dominated microbial population [48,49,50]. The lean–discriminant taxa comprised *Turicibacter*, *D. newyorkensis*, *Clostridia vadinBB60* group, *Bifidobacterium*, an unidentified *Bacilli* (RF_39unidentified) and *A. muciniphila*. Each of these taxa have been previously identified as inversely associated with obese phenotypes [51,52,53,54,55,56]. The utility of these taxa in promoting metabolic health directly has been explored via probiotic potential; supplementation with *D. newyorkensis* has demonstrated improved endothelial function in mice [57,58], and clinical trials with *A. muciniphila* have shown improvement in markers of MetS including insulin sensitivity and hypercholesterolemia [11,59,60].

With the exception of the *Clostridia vadinBB60* group, each of the lean–discriminant taxa demonstrated a significant negative correlation with body weight and/or OGT, supporting the previously reported effects of these organisms on metabolic regulation [61,62,63]. However, only *A. muciniphila* demonstrated a significant negative correlation with ROS-associated NIRF (Figure 3). While previous studies have shown that obese mice have greater levels of intestinal ROS compared to lean mice [16,18], and that *A. muciniphila* is inversely associated with the obese phenotype [64], our data demonstrate the first evidence of a direct relationship between relative abundance of *A. muciniphila* and the intestinal redox environment. This finding suggests that gut redox status may be a viable target in reversing dysbiosis and promoting metabolic health.

We next sought to determine whether *A. muciniphila* bloom is stimulated by reducing GI ROS regardless of the dietary radical scavenger, or if this phenomenon is exclusive to polyphenols. The dietary antioxidants of interest for this study, ascorbic acid, β-carotene and GPE were chosen based on previously determined radical scavenging efficacy directly upon administration [16] and known differences between physical characteristics and bioavailability (Table 1). The dose of antioxidant used in this study was chosen to remain consistent with previous studies from our research group which observed *A. muciniphila* bloom over the course of 14 days of treatment with grape polyphenols [9,43]. Based on allometric scaling, the dose of grape polyphenol extract used in this study is equivalent to 29.16 mg per kg body weight for a 60 kg adult [65]. Though these doses are higher than the estimated polyphenol intake in the average human diet (2 g/day) [66], previous studies have measured the scavenging of GI ROS at a lower concentration of antioxidants [16].

Of these antioxidant compounds, only GPE has previously been associated with stimulating *A. muciniphila* bloom [8,9,43]. The immediate effects of these compounds on GI ROS have been previously characterized, with GPE and β-carotene significantly decreasing GI ROS, and ascorbic acid, as a component of a mixture of other highly bioavailable antioxidant compounds, did not [16]. While useful in determining whether these dietary compounds are able to scavenge GI ROS, immediate imaging does not allow for evaluation of whether induced changes to the redox environment are sustained. A sustained reduction in GI ROS has been associated with improved glucose metabolism and modifications to the gut microbiome of obese mice [18]. Our findings demonstrated that, of the compounds tested, only GPE produced a sustained reduction in GI ROS 24 h after administration. Similarly, only treatment with GPE resulted in a significant percent increase in the relative abundance of *A. muciniphila* measured in the feces (Figure 4).

Our observation that relative abundance of *A. muciniphila* is inversely correlated with ROS-associated NIRF in lean mice treated with dietary antioxidants (Figure 4E) demonstrates an overarching benefit of dietary antioxidants in the support of a healthy gut microbiome, but the unique properties of GPE in comparison to ascorbic acid and β-carotene are further outlined using the differences observed in fecal antioxidant capacity (Figure 4F). Based on these findings, the poor bioavailability of GPE appears to be a benefit in comparison to other compounds, which are either absorbed in greater amounts or degraded over the course of digestion, thus preventing a sustained antioxidant effect in the GI lumen. 

The findings from this study have the potential for implications beyond MetS, as dietary polyphenols have also been shown to have immunomodulatory effects related to changes in the microbial ecology of the GI tract upon oral intake [67]. Considering the established links between oxidative stress and other chronic inflammatory conditions, the NIRF imaging described in this study may be a useful tool for the investigation of dietary antioxidants on other chronic inflammatory diseases, particularly those which primarily manifest in the GI tract such as inflammatory bowel disease or celiac disease. Further investigation into the role of intraluminal radical scavenging and modulation of the gut microbiome within the context of these conditions may lead to a better understanding of the biological mechanisms driving these conditions.

The present study is not without limitations; our design targeted proof-of-concept to identify the relationship between dietary antioxidants, the GI redox environment and the gut microbiome. As only lean mice were used in this study, future studies should investigate whether this mechanism also applies to obese mice where the initial abundance of *A. muciniphila* is often much lower, and where clear improvements in metabolic health (e.g., oral glucose tolerance) as a result of polyphenol treatment can be characterized alongside changes in the gut microbiome and redox environment. Additionally, inclusion of female mice would allow for the identification of differences between sexes and increase the potential for translatability to broader populations. Future research should also investigate longer-term interventions and the resilience of antioxidant-induced changes to the gut microbiome, as metabolic diseases tend to develop over long periods of time.

In order to increase the translatability of these findings towards dietary recommendations and meaningful improvements in human health, the research in this area will need to progress from preclinical models into clinical studies. Though the present study was designed specifically to provide mechanistic insight towards the relationship between dietary antioxidants and the GI redox environment, clinical trials would assist with translation of this concept into dietary recommendations. However, this may be challenging due to the limited options for reliable, non-invasive measurement of GI oxygen levels [68,69,70,71].

Overall, our findings suggest that the redox environment of the GI tract is a valuable therapeutic target for metabolic health and the reversal of dysbiosis. Though the strong correlations observed in this study do not definitively prove causality, they do identify possible therapeutic targets for the reversal of dysbiosis via dietary intervention and demonstrate that the administration of dietary antioxidants has the potential to modify the GI redox environment and support the growth of beneficial gut bacterium *A. muciniphila*. Furthermore, we demonstrate that not all antioxidants are created equal in this regard, as the efficacy with which *A. muciniphila* bloom is dependent on specific dietary antioxidants, with less bioavailable compounds yielding a more protective effect. Pre-absorptive mechanisms of poorly bioavailable dietary antioxidants warrant further exploration, especially with respect to chronic inflammatory diseases of the GI tract.

## Figures and Tables

**Table 1:** Solubility and bioavailability of dietary antioxidants used to scavenge GI ROS. Representative compounds for grape pomace extract are featured, as the extract was not characterized beyond total procyanidin B2 equivalents. Lower logP values indicate greater solubility in water. Superscript letters denote differences in experimental models for determining bioavailability.

Antioxidant Compound	LogP	Bioavailability
Ascorbic Acid	−1.6 [19]	76% ^1^ [20]
β-carotene	13.5 [21]	15.6% ^1^ [20]
Grape Pomace Extract		
Procyanidin B_2_	2.4 [22]	8–11% ^2^ [23]
Cyanidin-3-O-glucoside	0.39 [24]	12.4% ^1^ [25]
Quercetin	1.5 [26]	3.6% ^2^ [27]
Resveratrol	3.1 [28]	<1% ^1^ [29]

^1^ = study was conducted in humans; ^2^ = study was conducted in rats.

## Data Availability

16S rRNA amplicon sequencing reads from this data set have been deposited in the National Center for Biotechnology Information under BioProjectID PRJNA655513. Additional data presented in this study are available upon request to the corresponding author.

## Supplementary Materials

The following supporting information can be downloaded at https://www.mdpi.com/article/10.3390/antiox13030304/s1. Figure S1: Effect of dietary antioxidant treatment on phenotypic characteristics of lean mice. Figure S2: Effect of dietary antioxidant treatment on oral glucose tolerance of lean mice. File S1: Metadata, ASV Taxonomy and LefSe analysis results.
