# Supplementary material for: Dietary Polyphenols Support Akkermansia muciniphila Growth via Mediation of the Gastrointestinal Redox Environment
Source: Antioxidants (Basel). 2024 Feb 29;13(3):304. doi: 10.3390/antiox13030304 (PMC10967430; doi:10.3390/antiox13030304)
Supplement: Supplementary file 1 [file antioxidants-13-00304-s001.zip › 2024 - Van Butien et al - Supplementary Figures.pdf]

Supplementary Figures

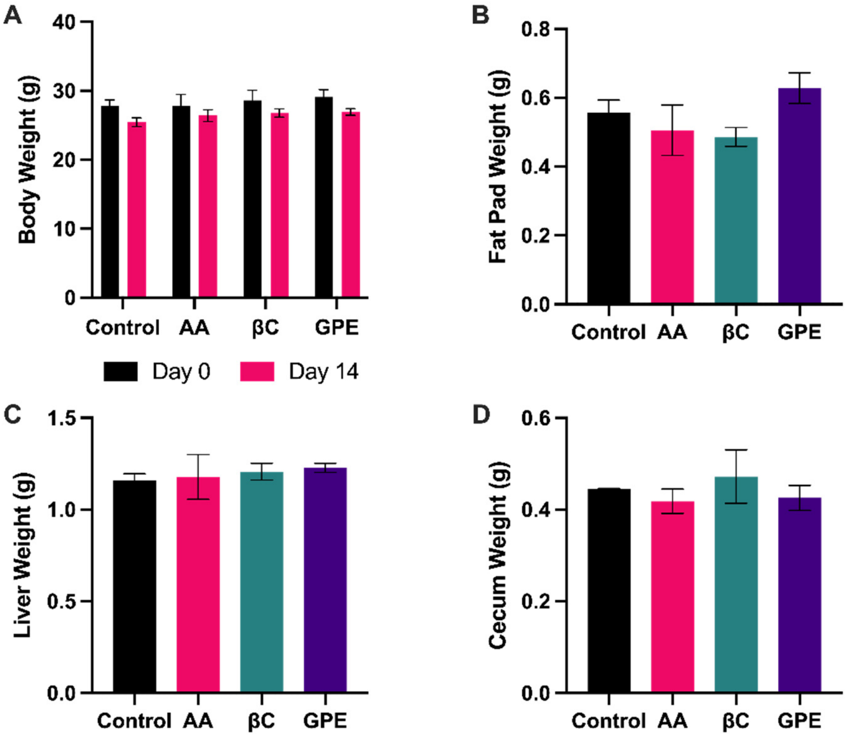

Figure S1. Effect of dietary antioxidant treatment on phenotypic characteristics of lean mice. Dietary antioxidant treatment did not affect (**A**) body weight, (**B**) fat pad weight, (**C**) liver weight or (**D**) cecum weight. Data were analyzed using two-way ANOVA with Tukey's multiple comparisons test and are reported as mean ± SD; $N = 5$ mice per group. AA = ascorbic acid; βC = β-carotene; GPE = grape polyphenol extract.

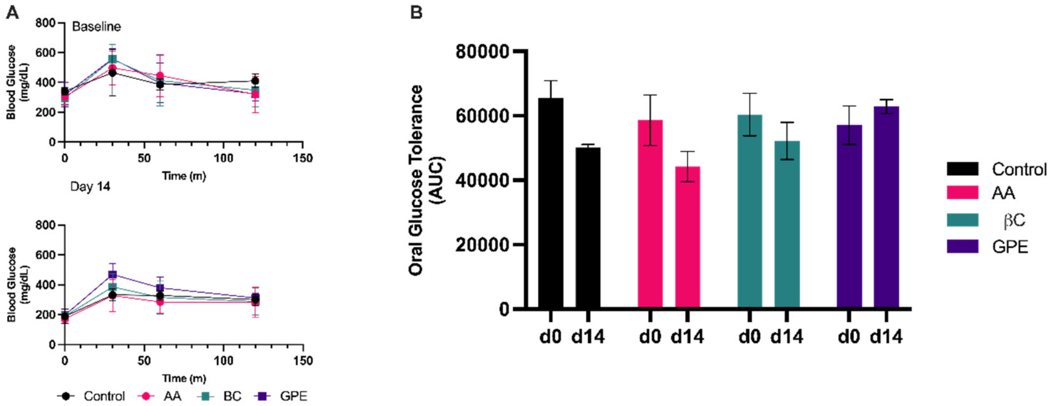

Figure S2. Effect of dietary antioxidant treatment on oral glucose tolerance of lean mice. Dietary antioxidant treatment did not affect oral glucose tolerance of lean mice over the course of 3 h. (**A**) Blood glucose concentrations (mg/dL) for mice over the course of 2 h. Data were analyzed using two-way ANOVA with Tukey's multiple comparisons test and are reported as mean ± SD; *N* = 5 mice per group. (**B**) Oral glucose tolerance expressed as area under the curve of blood glucose over the course of 2 h. Data were analyzed using two-way ANOVA with Tukey's multiple comparisons test and are reported as mean ± SD; *N* = 5 mice per group. AA = ascorbic acid; βC = β-carotene; GPE = grape polyphenol extract.
